# Collateral damage of the COVID-19 pandemic: an alarming decline in critical procedures in otorhinolaryngology in a German university hospital

**DOI:** 10.1007/s00405-020-06519-1

**Published:** 2020-12-15

**Authors:** Sarah Riemann, Iva Speck, Kathrin Gerstacker, Christoph Becker, Andreas Knopf

**Affiliations:** Department of Otorhinolaryngology—Head and Neck Surgery, University Hospital of Freiburg, Killianstraße 5, 79106 Freiburg, Germany

**Keywords:** Coronavirus, Head and neck surgery, Oncology, ENT, Cancer

## Abstract

**Purpose:**

The COVID-19 pandemic has a major impact on the diagnosis and treatment of ENT patients. The aim of this study was to analyze the influence of the pandemic on the number of otolaryngological procedures, particularly for critical diagnoses with potential negative effects due to prolonged symptom duration.

**Methods:**

We evaluated 10,716 surgical procedures between January 1, 2018 and May 31, 2020, focusing on the 16-week period around March 16, 2020, which includes 1080 observations. We further analyzed subsets of critical procedures.

**Results:**

We found a decline in critical procedures by 43% although no critical procedures were postponed by the hospital. Meanwhile, the share of critical procedures increased up to 90% caused by the cancellation of elective surgery. Especially worrisome was that diagnostic procedures for suspected malignancies decreased by 41% during the pandemic.

**Conclusion:**

The decline in critical procedures in otorhinolaryngology as collateral damage of the COVID-19 pandemic is considerable and therefore alarming.

**Supplementary Information:**

The online version contains supplementary material available at 10.1007/s00405-020-06519-1.

## Introduction

During to the outbreak of COVID-19, the treatment of ENT patients has largely suffered. Significantly reduced outpatient consultations and the discontinuation of elective surgery have far-reaching implications on the diagnosis and therapy of malignant and severe bacterial head and neck diseases. Because of the exposure to high viral load in patients’ upper airway mucosa and the large overlap of COVID-19 symptoms with other respiratory diseases, otorhinolaryngology has been in the spotlight of the COVID-19 pandemic [1, 2]. Otorhinolaryngologists have to carefully balance a uniquely high risk of life-threatening own infection against their indispensable contribution for the treatment of COVID-19-patients.

Most experts worry that the COVID-19 pandemic will negatively affect the treatment of cancer patients [3–5]. Particularly, the delay of treatment is known to have devastating effects on the functional outcome and on overall survival in head and neck cancer patients [6–8]. It was also recently shown that the proportion of patients with cancer was higher in the COVID-19 cohort than in the total population in China [9]. In addition, the risk of a severe course of the disease was increased in patients with the past and present oncological disease [9].

The collateral effect of the COVID-19 pandemic has been shown in only a few studies with quantitative data. Kasangra and Hamilton showed a decrease in stroke imaging in the United States by 39% during the early pandemic, a study from Austria showed a decline in acute coronary syndrome admissions and a survey from France demonstrated a drop of admissions to intensive cardiac care units under containment [10–12]. This evidence from other medical fields suggests that otorhinolaryngologic patients could also be neglected during the pandemic. Patients with initial diagnoses of malignancy or recurrence, but also with other critical diseases may not seek medical help or may seek it too late for fear of infection with COVID-19. At the same time, they are among the most vulnerable patient groups. In otorhinolaryngology, this concerns oncological diagnoses as well as acute inflammatory diseases, such as peritonsillar or neck abscesses and acute mastoiditis.

In the past weeks, a multitude of recommendations regarding ENT practice and surgery were published [13–16], but up to now there is no sufficient data allowing to assess the collateral effect of the pandemic on otorhinolaryngologic patients. The primary objective of the present study was to generate quantitative data of the influence of the COVID-19 pandemic on the number of critical surgical procedures in a large German ENT university hospital. The academic hospital Freiburg was at the epicenter of the COVID-19 pandemic in Germany due to its close proximity to Alsace (France) and North Italy and saw the highest numbers of COVID-19 patients. We aimed to estimate the collateral damage caused to ENT patients by the COVID-19 pandemic.

## Materials and methods

### Subgroup definition and data collection

We analyzed the data from all surgical procedures in the department of otorhinolaryngology, head and neck surgery, university hospital of Freiburg from January 1, 2018 until May 31, 2020. Patient data included age, gender, ICD-10 (International Classification of Diseases, Tenth Revision) diagnosis code(s) and the applicable OPS-codes, which is the German adaptation of the ICPM (International Classification of Procedures in Medicine) [17]. Procedures with missing data (< 0.2%) were excluded from the analysis. The full data set consisted of 10,716 observations. The 16-week period around March 16, 2020 contained 1080 observations.

From the complete catalogue of OPS codes, we defined a list of critical otorhinolaryngological procedures (Table 2, Supplementary Tables). Within the critical procedures, we further defined subcategories: ‘Suspected malignancy’ indicates procedures that are performed to confirm potential malignancy (e.g., diagnostic lymph node excision or pharyngo-/laryngoscopy), ‘tumor operation’ for surgery of head and neck cancer, ‘salivary gland surgery’ for any procedures of masses in the submandibular or parotid gland and ‘emergency procedures’ for procedures which needed to be performed in a time frame of up to 24 h. These ‘emergency procedures’ were further categorized in procedures for acute bleeding (e.g., epistaxis) and for acute inflammatory diseases like neck abscesses (e.g., peritonsillar abscess, para-/retropharyngeal abscess) and acute mastoiditis.

The present study was approved by the local ethics committee of Freiburg University (EK Freiburg, 302/20) and registered at the German Clinical Trials Register (DRKS00021699).

### Statistical analysis

March 16, 2020 was chosen as the date to separate an ‘eight-week-before’ (January 20 until March 15, 2020) and ‘eight-week-after’ period (March 16 until May 10,2020), because it was the date that a Government Decision from March 12, 2020, cancelling all elective surgical procedures and inpatient treatment, went into effect. For the corresponding periods in 2018 and 2019, weeks were defined as seven-day intervals around March 16, not necessarily starting on Mondays. The distribution of German public holidays (e.g., Easter) across ‘before’ and ‘after’ periods did not differ between the 3 years. Percentage decline was calculated comparing the total count of procedures of the ‘before March 16, 2020′ period and the ‘after March 16, 2020′ period. All *p* values were determined using the Wilcoxon–Mann–Whitney test. A level of significance of 0.05 was applied in all analyses. All analyses were performed with R (version 3.6.3).

## Results

### Surgical procedures and patients’ characteristics

We evaluated data of 10,716 surgical procedures between January 1, 2018 and May 31, 2020. The 16-week period around March 16, 2020 consisted of 1080 observations of which 543 procedures were classified as being critical. Comparing the’before March 16′ and ‘after March 16′ periods, the percentage of female patients of all critical cases remained nearly unchanged at 39 and 40% respectively (Table 1). However, the age differed significantly between the two periods: patients in the ‘after March 16′ group were significantly older (Table 1) with a median age of 45 years before March 16, 2020 and 49 years after March 16, 2020.

**Table 1:** Age and gender of critical cases between January 20 and May 10, 2020

	Before March 16	After March 16	*p* values
Age	45.17 (43.83, 46.86)	49.02 (46.92, 50.67)	0.015
Share of female patients	0.38 (0.36, 0.43)	0.39 (0.33, 0.50)	1

All data are reported as median and first and third quartiles.* p* values of Wilcoxon–Mann–Whitney tests

**Fig. 1:**
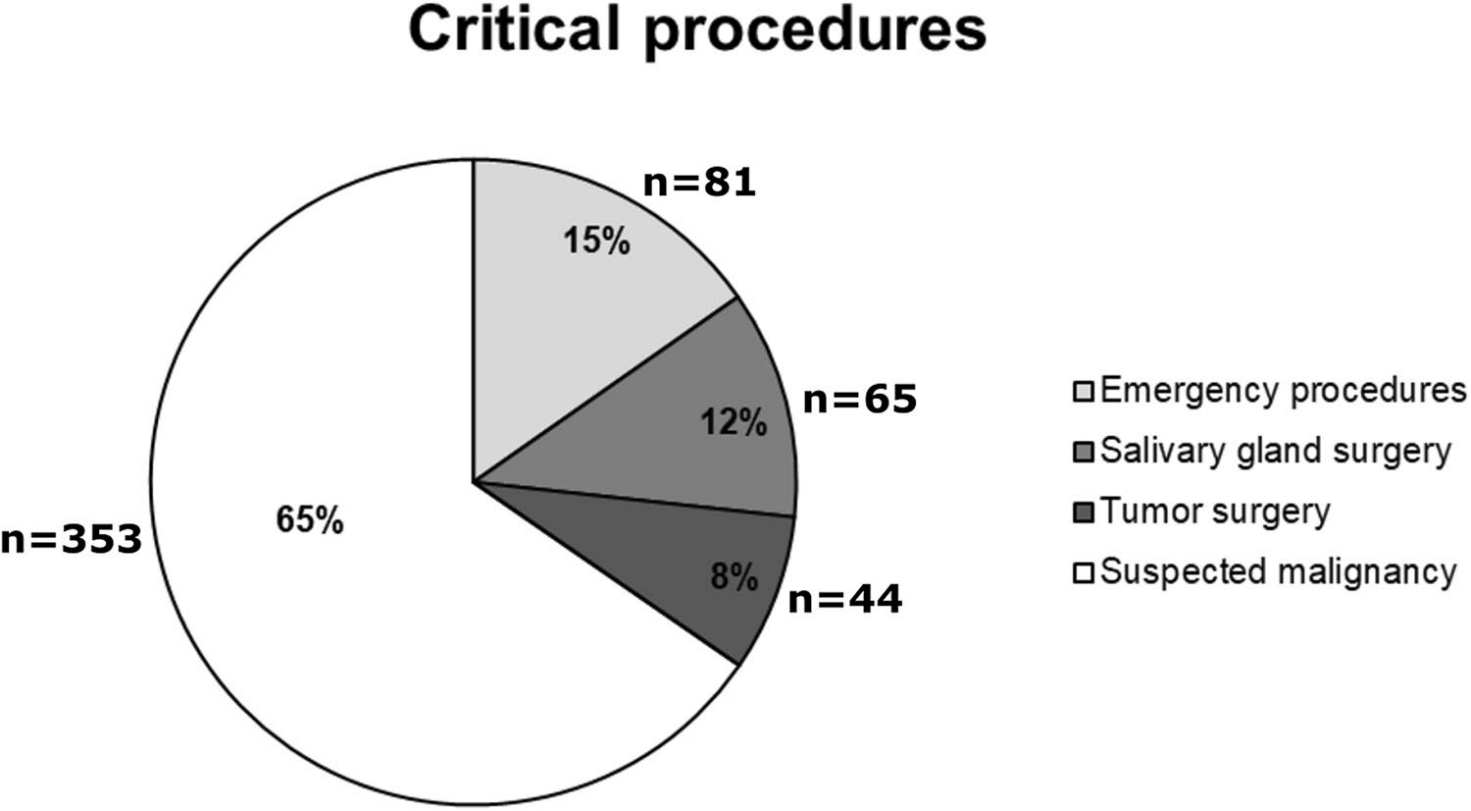
Subcategory percentage and total numbers of all critical procedures between January 20 and May 10, 2020

We defined four subgroups of critical procedures. Critical procedures were for suspected malignancy in 65%, for emergencies in 15%, salivary gland surgery in 12%, and for tumor operations in 8% of all critical cases treated between January 20 and May 10, 2020 (Fig. 1). Acute inflammatory diseases caused 75% of the emergency procedures, while one quarter was because of acute bleeding. These percentages did not change significantly during the COVID-19 pandemic (data not shown).

### Effect of the COVID-19 pandemic

**Fig. 2:**
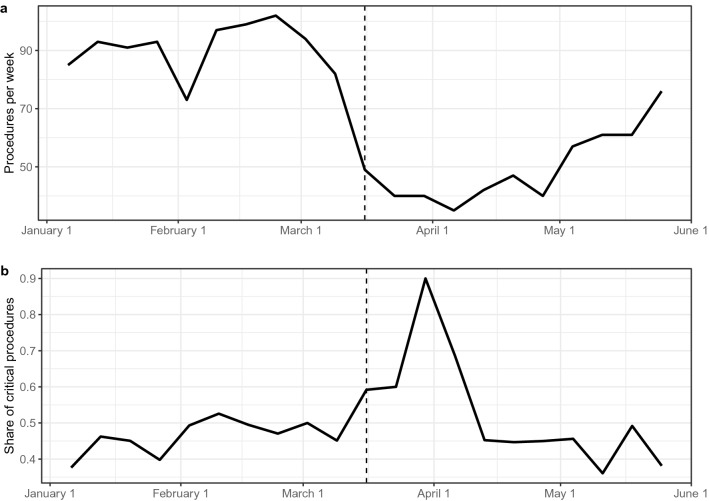
Total number of procedures per week (**a**) and share of critical procedures per week (**b**) from January 6 until May 31, 2020. Dashed line indicates March 16, 2020

On March 16, 2020, a governmental decision went into effect cancelling all elective surgical procedures in Germany [18], which decreased the total number of procedures in our department (Fig. 2a). At the same time, the share of critical procedures drastically increased (Fig. 2b). Although the percentage of critical procedures was between 40 and 55% in the first 10 weeks of 2020, it went up to 90% in the last week of March after the governmental decision. As the number of total procedures in our department increased again, the share of critical cases declined to its initial level (Fig. 2a, b).

**Fig. 3:**
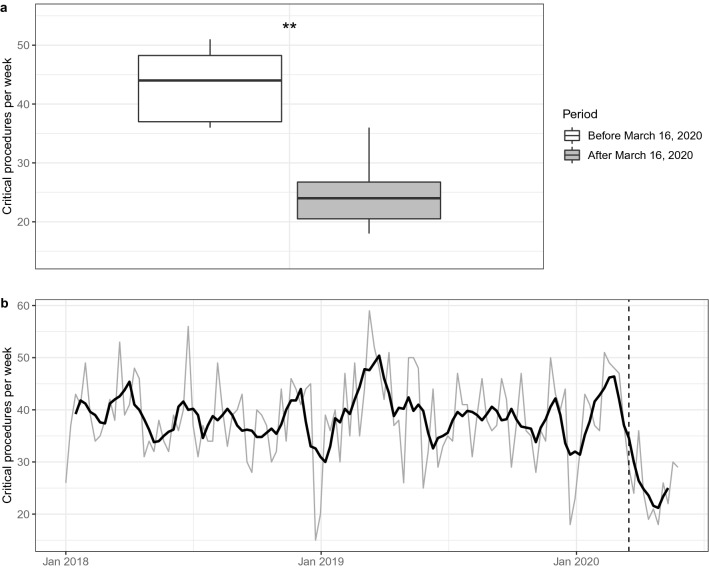
Number of weekly procedures eight weeks before and eight weeks after March 16, 2020 (**a**) and number of critical procedures per week (grey line) and 5-week moving average (black line) from January 1, 2018 until May 31, 2020 (**b**). Dashed line indicates March 16, 2020. Comparison between groups was done using a Wilcoxon–Mann–Whitney test. **p < 0.01

Although critical procedures were exempt from regulation and no critical procedures were cancelled, we still observed a dramatic drop, cutting the number of treated critical cases almost in half. The median number of critical procedures per week fell significantly from 44 before to 24 after March 16, 2020 (Fig. 3a, Table 3 and 4, Supplementary Tables). Similarly, the total number of critical procedures decreased by 43%.

To put this effect size into perspective, the immediate drop is comparable to the decrease of critical procedures seen every year during the Christmas holidays (Fig. 3b). However, the lower surgical volume around Christmas only last up to 10–14 days, while the effect of the COVID-19 pandemic on critical procedures is sustained over several weeks until the end of May, the end of our study period.

**Fig. 4:**
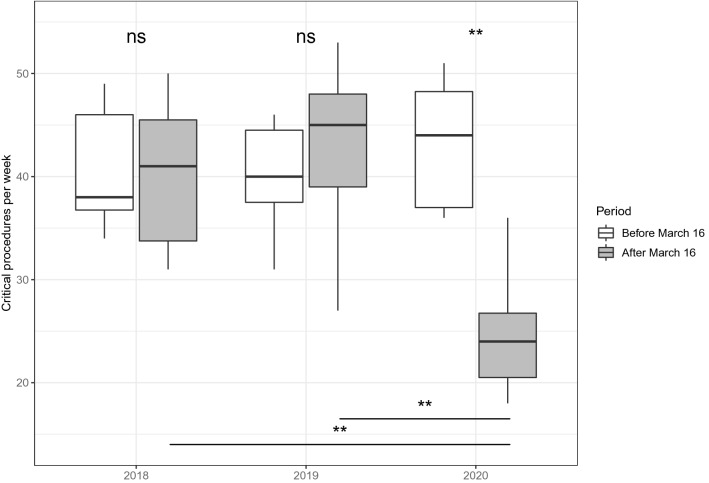
Number of weekly procedures 8 weeks before and 8 weeks after March 16, 2018, March 16, 2019, and March 16, 2020, respectively. Significance levels of the comparison within a year are shown above and comparison of the ‘after’ period between 2018 and 2019 with 2020 are shown below the boxplots. Comparisons between groups were done using a Wilcoxon–Mann–Whitney test. **p < 0.01

To further analyze whether this drop could be due to seasonal effects, we compared our critical time period to the corresponding 8-week periods of the two previous years. A significant decrease in numbers of critical procedures was also found when comparing the ‘after March 16′ periods in 2020 with the respective periods in 2018 and 2019. Also, the comparison between the ‘before March 16′ and ‘after March 16′ periods within earlier years revealed no significant difference in number of critical procedures in 2018 and 2019 (Fig. 4, Tables 3 and 4, Supplementary Tables).

**Fig. 5:**
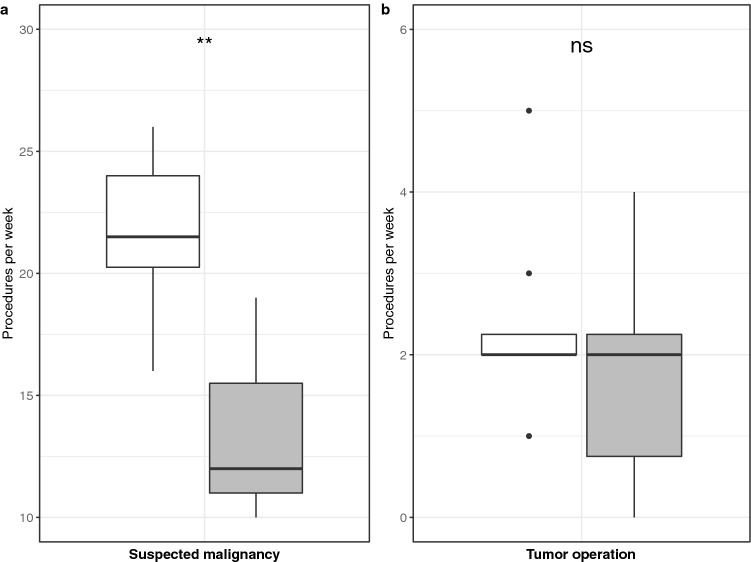
Number of weekly procedures eight weeks before and eight weeks after March 16, 2020. There was a decrease in procedures for suspected malignancies (**a**), while the number of cancer surgeries per week did not significantly change (**b**). Comparisons between groups were done using a Wilcoxon–Mann–Whitney test. **p < 0.01

To explore possible causes and evaluate the resulting implications of our results, we analyzed the effects of the COVID-19 pandemic on the four subgroups of critical procedures. We found a significant decrease in procedures for diagnosis of suspected malignancy from a median number of 22 per week to 12 (Fig. 5a, Tables 3 and 4, Supplementary Tables). Correspondingly, the total number of critical diagnostic procedures dropped by 41% comparing the ‘before March 16, 2020′ and ‘after March 16, 2020′ periods. When analyzing emergency procedures for acute bleeding and acute inflammatory diseases, we found a downward trend by 51%, although this was barely not statistically significant compared to the 8 weeks before (*p* = 0.054). The number of tumor operations did not change significantly (Fig. 5b, Table 3 and 4, Supplementary Tables) and neither did procedures for salivary gland masses (Tables 3 and 4, Supplementary Tables).

## Discussion

We demonstrate a dramatic drop in the number of diagnostic and therapeutic procedures for severe bacterial and malignant head and neck diseases. Our methodology does not only assess the impact of COVID-19 within 2020, but also compares it to prior years, which gives us stronger and more robust results. The present study reveals that critical surgical interventions significantly decreased even though they were not affected by the government intervention or cancelled by the hospital. This decline in otorhinolaryngological procedures mirrors the decrease found in similar studies on stroke imaging, acute coronary syndrome admissions, and admissions to intensive cardiac care units [10–12]. Although many hypotheses might explain such effects, we have to assume that patients’ fear of SARS-CoV-2 infection during their clinical stay is one of the main causes [19–21].

Numerous otolaryngological publications discuss the high risk of ENT procedures and emphasize the need for comprehensive guidelines [22–24]. In practice, Kuhar et al. reported an extremely high percentage of aerosol-generating otolaryngological procedures in Ohio [25]. Furthermore, a few studies documented the development of patient numbers during the COVID-19 pandemic, but mostly lack in-depth statistical analysis. Kuhar et al. found a large drop in surgical procedures in the United States, but did not study further subcategories [25]. Ralli et al. demonstrated a decrease in operative procedures mainly driven by a drop in procedures for abscesses and nasal bone fractures in Italy, while numbers of cancer surgery remained stable [26]. This contrasts our findings and is most likely due to the absence of statistical analysis and considerably lower number of cases, which is less representative and sensitive to smaller changes.

The sharp drop in procedures for diagnosis of malignancy by almost half is the most worrisome finding of our study. The potentially grave effects of the COVID-19 pandemic on cancer patients have also been shown by Sud et al., who modeled a per-patient delay of surgery of 3 or 6 months and found significant impact on survival for various cancer types [27], but only in a theoretical setting. Just recently, Schutte et al. showed an increased 3-year overall survival by 12% after shortening the diagnosis-to-treatment interval of head and neck cancer patients by only 13 days [28]. However, the delay of diagnosis and treatment for our patients during the COVID-19 pandemic is most likely even longer than 13 days. These studies, combined with our findings, confirm the initial fears that the COVID-19 pandemic would negatively affect the treatment of cancer patients [3–5].

Furthermore, the discontinuation of routine follow-up appointments for head and neck cancer patients during the COVID-19 pandemic could additionally cause the decline in diagnostic procedures and could result in a longer interval until the diagnosis of a recurrence. Patients whose follow-up appointment was postponed could discontinue their regular visits altogether so it’s imperative to keep close track of these patients.

Although the decline in the subgroup of emergency procedures is not significant, most likely due to the small number of weekly cases, the effect is still interesting because of its large size. The most likely explanation is a reduced spread of respiratory pathogens due to social distancing measures and stay-at-home orders. This hypothesis is also consistent with our statistical findings of an overall large drop but no sharp decline: In contrast to the sudden drop in surgical procedures due to a single government intervention, the introduction of social distancing measures was a more gradual process.

Alternatively, it is possible that these patients simply also try to avoid consulting a physician due to fear of COVID-19 infection, they might do so less often because of the urgency of their symptoms.

In conclusion, the repercussions of the COVID-19 pandemic have affected and will continue to affect otorhinolaryngologic patients in the coming months. Precise estimations of the extent of negative effects on functional and oncological outcome should be object of further investigation.

## Supplementary Information

Below is the link to the electronic supplementary material.

Supplementary file1 (PDF 390 KB)
