# Supplementary material for: Collateral damage of the COVID-19 pandemic: an alarming decline in critical procedures in otorhinolaryngology in a German university hospital
Source: Eur Arch Otorhinolaryngol. 2020 Dec 15;278(9):3417–23. doi: 10.1007/s00405-020-06519-1 (PMC7736669; doi:10.1007/s00405-020-06519-1)
Supplement: Supplementary file 1 — Supplementary file1 (PDF 390 KB) [file 405_2020_6519_MOESM1_ESM.pdf]

European Archives of Oto-Rhino-Laryngology

Sarah Riemann[1], Iva Speck[1], Kathrin Gerstacker[1], Christoph Becker[1], Andreas Knopf[1]

Collateral damage of the COVID-19 pandemic – an alarming decline in critical procedures in otorhinolaryngology in a German university hospital

[1] Department of Otorhinolaryngology - Head and Neck Surgery, University Hospital of Freiburg, Germany

Corresponding author:

Sarah Riemann

Department of Otorhinolarnygology- Head and Neck Surgery

University Hospital of Freiburg

Killianstraße 5, 79106 Freiburg, Germany

sarah.riemann@uniklinik-freiburg.de

Table 2. OPS-Codes used to define 'critical' procedures, with subcategories 'suspected malignancy', 'emergency', 'salivary gland surgery' and 'tumor surgery'.

Category	Subcategory	OPS-code
Critical	Suspected malignancy	5-401.00, 1-610.0, 1-611.0, 1-630.1
Critical	Emergency	5-210.1, 5-210.4, 5-289.1, 5-289.2, 5-211.20, 5-270.5, 5-270.6, 5-281.1, 5-280.0, 5-280.1, 5-280.2, 5-280.3, 5-280.x, 5-203.0*
Critical	Salivary gland surgery	5-262.00, 5-262.01, 5-262.02, 5-262.03, 5-262.04, 5-262.05, 5-262.0x, 5-262.11, 5-262.12, 5-262.13, 5-262.14, 5-262.15, 5-262.1x, 5-262.20, 5-262.21, 5-262.22, 5-262.23, 5-262.24, 5-262.25, 5-262.2x, 5-262.3, 5-262.40, 5-262.41, 5-261.3, 5-261.4

* Mastoidectomy was only considered ‘critical’ in combination with the ICD-10 code for acute mastoiditis, (H70.0).

Category	Subcategory	OPS-code
Critical	Tumor surgery	5-251.00, 5-251.01, 5-251.02, 5-251.03, 5-251.0x, 5-251.10, 5-251.11, 5-251.12, 5-251.13, 5-251.1x, 5-251.20, 5-251.21, 5-251.22, 5-251.23, 5-251.2x, 5-251.x0, 5-251.x1, 5-251.x2, 5-251.x3, 5-251.xx, 5-252.00, 5-252.01, 5-252.02, 5-252.03, 5-252.0x, 5-252.10, 5-252.11, 5-252.12, 5-252.13, 5-252.1x, 5-252.20, 5-252.21, 5-252.22, 5-252.23, 5-252.2x, 5-252.30, 5-252.31, 5-252.32, 5-252.33, 5-252.3x, 5-252.40, 5-252.41, 5-252.42, 5-252.43, 5-252.4x, 5-252.x0, 5-252.x1, 5-252.x2, 5-252.x3, 5-252.xx, 5-277.00, 5-277.01, 5-277.02, 5-277.03, 5-277.0x, 5-277.10, 5-277.11, 5-277.12, 5-277.13, 5-277.1x, 5-277.20, 5-277.21, 5-277.22, 5-277.23, 5-277.2x, 5-277.30, 5-277.31, 5-277.32, 5-277.33, 5-277.3x, 5-277.x0, 5-277.x1, 5-277.x2, 5-277.x3, 5-277.xx, 5-281.2, 5-295.00, 5-295.01, 5-295.02, 5-295.03, 5-295.04, 5-295.05, 5-295.0x, 5-295.10, 5-295.11, 5-295.12, 5-295.13, 5-295.14, 5-295.15, 5-295.1x, 5-295.30, 5-295.31, 5-295.32, 5-295.33, 5-295.34, 5-295.35,

	5-295.3x, 5-295.x0, 5-295.x1, 5-295.x2, 5-295.x3, 5-295.x4, 5-295.x5, 5-295.xx, 5-296.00, 5-296.01, 5-296.02, 5-296.03, 5-296.04, 5-296.05, 5-296.06, 5-296.07, 5-296.0x, 5-296.10, 5-296.11, 5-296.12, 5-296.13, 5-296.14, 5-296.15, 5-296.16, 5-296.17, 5-296.1x, 5-296.30, 5-296.31, 5-296.32, 5-296.33, 5-296.34, 5-296.35, 5-296.36, 5-296.37, 5-296.3x, 5-296.x0, 5-296.x1, 5-296.x2, 5-296.x3, 5-296.x4, 5-296.x5, 5-296.x6, 5-296.x7, 5-296.xx, 5-301.0, 5-301.1, 5-301.2, 5-301.3, 5-301.x, 5-302.0, 5-302.1, 5-302.2, 5-302.3, 5-302.4, 5-302.5, 5-302.6, 5-302.7, 5-302.8, 5-302.9, 5-302.x, 5-303.00, 5-303.01, 5-303.02, 5-303.03, 5-303.04, 5-303.05, 5-303.06, 5-303.07, 5-303.0x, 5-303.10, 5-303.11, 5-313.12, 5-313.13, 5-313.14, 5-313.15, 5-313.16, 5-313.17, 5-313.1x, 5-303.20, 5-303.21, 5-323.22, 5-323.23, 5-323.24, 5-323.25, 5-323.26, 5-323.27, 5-323.2x, 5-303.x0, 5-303.x1, 5-303.x2, 5-303.x3, 5-303.x4, 5-303.x5, 5-303.x6, 5-303.x7, 5-303.x

Table 3. P-values of Wilcoxon-Mann-Whitney tests.

			p-value
Critical_2018_before	vs	Critical_2018_after	0.71
Critical_2019_before	vs	Critical_2019_after	0.56
Critical_2020_before	vs	Critical_2020_after	0.001
Critical_2018_after	vs	Critical_2020_after	0.003
Critical_2019_after	vs	Critical_2020_after	0.002
Suspected malignancy_2020_before	vs	Suspected malignancy_2020_after	0.002
Tumor operation_2020_before	vs	Tumor operation_2020_after	0.43
Emergency procedures_2020_before	vs	Emergency procedures_2020_after	0.05
Salivary gland surgery_2020_before	vs	Salivary gland surgery_2020_after	0.05

Table 4. Weekly number of respective procedures eight weeks before and after March 16. All data are reported as median and first and third quartiles.

	Before March 16	After March 16
**Year 2020**		
Critical	44 (37, 48.25)	24 (20.25, 26.75)
Suspected malignancy	21.5 (20.25, 24)	11.5 (10.75, 15.25)
Tumor operation	2 (2, 2.25)	2 (0.75, 2.25)
Emergency procedures	4.5 (3, 6.75)	3 (1.75, 3.25)
Salivary gland surgery	3 (2.75, 4.25)	3 (1.75, 3.25)
**Year 2019**		
Critical	42 (37.75, 45.25)	45 (39, 48)
**Year 2018**		
Critical	38 (36.75, 46)	41 (33.75, 45.5)
